# Supplementary material for: Are carbon emissions trading and green financial instruments synergistic? -Comprehensive quantitative research based on content analysis
Source: PLoS One. 2024 Mar 7;19(3):e0298601. doi: 10.1371/journal.pone.0298601 (PMC10919601; doi:10.1371/journal.pone.0298601)
Supplement: S1 Data — (ZIP) [file pone.0298601.s001.zip › Data/地级市Z_F工作报告.zip!/┴╔─■/╕╖╨┬╩╨/└┤╫╘╣1⁄2╓┌║┼í╛┬φ┐╦╩2╛▌═°í┐_2017.docx]

各位代表：

　　现在，我代表市政府向大会报告工作，请予审议，并请市政协委员和其他列席同志提出意见。

　　一、2016 年工作回顾

　　过去一年，在省委、省政府和市委的正确领导下，在市人大依法监督和市政协民主监督下，我们牢固树立新发展理念，全面贯彻“四个着力”要求，坚持稳中求进总基调，统筹稳增长、促改革、调结构、惠民生、防风险，推动全市经济社会平稳发展。

　　预计完成地区生产总值450亿元；固定资产投资87 亿元；一般公共预算收入35.8亿元；城镇、农村常住居民人均可支配收入分别实现24040 元、11720 元，分别增长6%、5.5%。过去一年的经济发展，在坚持实事求是的基础上，更加注重转换新旧动能，更加注重提升质量效益，更加注重改革创新驱动，更加注重守住民生底线，为全市经济筑底向好、稳步增长奠定了坚实基础。

　　一年来，我们主要抓了以下六个方面工作：

　　（一）全力以赴稳增长，经济运行总体平稳

　　项目建设势头良好。深入开展“项目年”活动，实施市政府班子“四个一”等工作机制，全年新开工投资亿元以上项目44个、竣工投产五千万元以上项目125个。大唐煤制天然气项目复工准备就绪，远东铜业、宝厦集成建筑等项目部分投产，万达广场、红星国际广场等项目主体完工，德尔二期、华禹冶金、天士力、兴隆CBD三期等项目加快建设，富国皮革总部搬迁、大鹰组建集团等一批项目加快实施。争取中央预算内资金5.3亿元、国家专项建设基金2.4亿元、省产业引导基金1.2亿元。

　　对外开放成效明显。坚持领导带头招商，先后赴京津冀、长三角、珠三角等地招商40余次、推进项目130余个，保利生产线智能化改造等30余个项目签约，中国华录文化旅游、国药集团等大型央企、民企融合取得实质进展。精心谋划“请进来”活动60 多次，同各地商会、协会招商合作不断深化。承接上海和北京通州产业转移积极推进。新增出口企业15 户，出口额2.1 亿美元。成功举办第四届中国氟化工论坛，参加广交会等国际展会11次。

　　企业运行保持平稳。深入开展“企业服务年”活动，加强企业运行监测，坚持月调度、季分析，市级领导包保企业帮助解决问题1000 余个。大金、伊利、鲁花、力劲等40户骨干企业运行良好，55户规上企业恢复生产。超懿集团并购重组域外企业进展顺利。阜矿集团白音华井工矿开工建设，转移人员5000 人。市矿合作不断深化。为126户中小企业担保贷款19.2亿元。

　　消费拉动作用增强。成功举办玛瑙、房地产、皮革、汽车、建材家居等大型展会13次，开展大型促销活动20 余次。社会消费品零售总额实现283亿元，增长3%。

　　（二）坚定不移调结构，转型升级基础夯实

　　制造强市战略深入实施。编制了中国制造2025 阜新行动纲要和八大类工业产业发展方案，实施智能化、数字化车间改造项目30 个，重点对液压装备等产业集群改造升级。氟产业“智慧园区”列入省首批唯一示范园区。超懿集团产品荣获中国驰名商标。页岩气勘探开发取得积极进展。

　　现代农业迈上新台阶。沈阜现代农业示范带四年任务三年完成，累计土地流转163 万亩、建设面积208 万亩、实施项目479 个，核心区土地亩收益是传统农业的6.4 倍。新益达羊毛加工、中澳合资奥博生物等项目竣工投产，天津金地果蔬食品、辉山肉牛屠宰等项目加快建设。调减玉米面积32.9万亩，粮食产量41 亿斤。新建改扩建标准化生态养殖小区51个。新增节水灌溉8万亩。柳河治理一期工程完工。农田作业机械化水平达到87.3%。发展农民专业合作社729家。完成农村土地确权272万亩。国家农村信息进村入户试点全省领先。祥和牧业秸秆膨化饲料、惠农生物质热电项目正式运行，秸秆综合利用走在全省前列。

　　现代服务业持续壮大。6个省级服务业集聚区实现营业收入320亿元，增长10%。紫金财富广场、香港豪德二期、亚欧国际建材城等专业市场投入运营，邮政集团阜新物流中心开展前期工作。章古台沙地国家森林公园获批，阜蒙县成功创建省级旅游产业发展示范县，温泉旅游度假区年接待游客60万人次，黄家沟生态旅游服务区成为近郊旅游新热点。全市银行机构人民币各项存款余额突破千亿元。天佐公司在“新三板”挂牌上市，阜新云想文化等3户企业在上海股权交易中心挂牌，阜新银行、金凯化工等一批企业上市进展顺利。新增证券、保险等金融机构3家。中国网库、颐高集团等电商领军企业落户。

　　（三）扎实稳妥促改革，发展活力有效释放

　　重点领域改革取得实效。政府机构改革有序开展。推进行政审批事项“两集中、两到位”，成立了市行政审批服务局。深化“放管服”改革，市级行政审批事项减少25项。纳入权责清单职权1625项。33家经营性事业单位转企改革方案、虎跃集团重组市公交公司、2户央企“三供一业”职能移交、机关公务用车改革等工作完成。交通、城建、旅游3个企业集团成立。公共资源服务系统和电子交易平台投入使用。市公共服务事项目录及办事指南公布。供热资源整合工作启动。出台了优化发展环境意见。实行了“五证合一、一照一码”，新增各类市场主体1.2万户。工业用地市场化配置、不动产统一登记等工作稳步推进。“营改增”试点全面推开。首批国有林场改革方案获省批复。

　　供给侧结构性改革扎实推进。“三去一降一补”工作深入开展。2户地方煤炭企业关闭，统筹协调阜矿集团去产能涉及的地方工作；出台18条政策，去化商品房库存65万平方米；规范管理小贷公司，有效防范区域性金融风险，引导金融机构支持实体经济；兑现国家和省涉企优惠政策，为企业减负1亿多元；战略新兴产业、精准脱贫、公共服务等方面短板得到加强。

　　创新驱动战略稳步实施。德尔公司创建全市首个国家级技术中心，新增省级企业中心3个、工程技术研究中心2个，申请国际专利6件。市校合作深入开展，建立辽宁工大、氟化工产业基地等13家“众创空间”。成立全国首个花生产业院士工作站。设立产业引导基金，推出PPP项目42个。实施科技成果转化30项，培育创新型企业10户，扶持创业带头人506人。

　　（四）坚持不懈抓生态，城乡环境不断改善

　　生态环境持续提升。海州露天矿南帮治理一期工程通过省验收，全省首个境外低息——德国5000万欧元贷款项目获批；细河水质提升、污水处理设施建设、城市防汛“3+1”工程前期工作等进展顺利。拆除燃煤小锅炉245台，淘汰黄标车及老旧车8476辆。清理整顿环保违规项目1289个。宜居乡村建设扎实推进，硬化村屯道路900公里。人工造林20万亩，草原生态治理3万亩，跨区域防沙治沙综合示范区建设取得明显成效。环境空气质量达标率83%。

　　基础设施建设增强。京沈客专阜新段铺架轨道45公里，彰武段联接线开工。巴新铁路货运量达200万吨。阜盘铁路、阜奈高速前期工作稳步推进。500千伏变电站开工建设。改造供水、供热、供气管网221公里，城区天然气转换完成。改造街路17条，新建、改造农村公路404公里。创业一街跨细河桥通车，青年街桥主体完工。数字化城市管理系统初步建立。

　　（五）千方百计惠民生，社会事业协调发展

　　就业和社会保障保持稳定。全市城镇新增就业1.7万人。企业基本养老金月人均增加133元。城市低保月标准达到490元，农村低保年标准达到3264元。新农合实现全覆盖，年人均补助标准提高40元；城镇居民医疗保险年人均补助标准提高60元。职工月最低工资标准提高到1200元。残疾人“两项补贴”政策全面推进。劳动关系保持和谐。

　　脱贫攻坚深入开展。精准扶贫“五个一批”扎实推进，全市9.6万贫困人口全部建档立卡，当年3.5万人脱贫，66个贫困村脱贫“销号”，海州、细河、太平贫困人口全部脱贫。

　　保障性安居工程扎实推进。棚改开工777套，筹集公租房1516套，新增廉租房租赁补贴500户。投放住房公积金贷款及提取资金13亿元。改造农村危房3296户。

　　社会事业全面发展。阜蒙县、海州区、太平区义务教育均衡工作通过国家认定。成功荣获“世界玛瑙之都”称号；以董福财为原型的电影《愚公移山》列入全国百个孵化项目。公立医院改革稳步推进，基本药物制度全面实施；基本公共卫生服务年人均经费提高到45元。“全国全民健身示范市”成果得到巩固。市三院改扩建、妇女儿童活动中心等工程主体完工，市蒙中教学楼、一职专实训楼等工程投入使用，市中心血站、卫生监督所综合楼加快建设。民族团结进步事业不断发展，宗教工作和谐稳定。圆满完成村委会换届工作。第三次全国农业普查取得阶段性成果。成功创建第八轮全国双拥模范城。

　　各项风险有效防范。深入贯彻落实辽委发19号、21号文件精神，完善了偿债准备金制度，通过地方债券置换、基金整合等方式，有效化解政府债务；制定16项措施，强化城镇企业基本养老保险征缴、社保扩面工作，确保了按时足额发放。安全生产形势总体稳定。食品药品监管能力增强。领导干部接访深入推进。应急管理得到加强。社会治安立体化防控网络更加完善，一批重大刑事案件、命案积案成功侦破，人民群众安全感和满意度进一步提高。

　　（六）真抓实干转作风，政府自身建设得到加强

　　全面落实党建责任。深入学习贯彻十八届六中全会精神，严格执行《准则》和《条例》要求，坚定不移地同以习近平同志为核心的党中央保持高度一致。深入开展“两学一做”学习教育，全面落实“一岗双责”。以辽宁拉票贿选案为镜鉴，净化政治生态。认真抓好国家和省巡视组、督查组意见整改落实。市政府党组全年政治理论学习19次，自觉践行市委常委会作出的“九个坚持、九个自觉”公开承诺。

　　全面执行上级决策。深入贯彻落实中发7号和国发28号、62号文件精神，印发了任务分解和责任分工，出台了新一轮转型振兴三年滚动计划，正在编制转型升级行动计划。向省政府和市委报告工作60余次，学习传达贯彻省委、省政府各类会议40余次。制定出台壮大战略性新兴产业等政策文件55个。

　　法治政府建设加强。自觉接受市人大法律监督和市政协民主监督，建议提案办复率100%。出台法治政府建设实施方案，建立了重大行政决策程序规定，实现部门、县区法律顾问全覆盖。加强对养老、低保、医保等重点民生资金的审计监督。行政监察力度加大。建立“三重一大”审议机制，市政府党组议事制度不断完善。

　　外事、侨务、对台、市志、档案、青年、妇女儿童、残疾人事业、红十字、老龄、慈善、人防等工作取得新成绩，国税、地税、消防、电力、邮政、通信、石油、烟草等工作取得新进展。

　　各位代表！全市经济社会发展取得的成绩，是省委、省政府亲切关怀、大力支持的结果，是市委总揽全局、正确领导的结果，是市人大、市政协认真履职、有效监督的结果，是全市各族人民齐心协力、不懈奋斗的结果。在此，我代表市人民政府，向全市各族人民，向市人大代表、政协委员，向各民主党派、工商联、无党派人士和各人民团体，表示诚挚感谢！向参与阜新建设发展的中省直单位，向解放军驻阜部队、武警官兵，向所有关心、支持阜新发展的同志们、朋友们，表示诚挚感谢！

　　在总结工作的同时，我们也看到存在的问题：一是结构调整、产业升级要求紧迫，供给侧结构性改革还需加强，新旧动能转换任务艰巨；二是受宏观经济形势和阜矿集团去产能等方面影响，工业经济下行压力增大；三是创新创业氛围不浓、水平不高，经济发展软环境需要切实改善，体制机制创新步伐还需加快；四是生态环境仍然脆弱，环境保护治理任务艰巨；五是历史遗留问题和现实问题相互交织，财政收支矛盾突出，防范政府性债务和养老金风险任务较重，保障和改善民生压力巨大；六是政府系统个别干部与新常态的要求不相适应，不同程度存在主观不努力、不担当、不作为、不创新、工作落实不力等现象。这些问题必须引起高度重视，在今后工作中切实加以解决。

　　二、2017 年总体要求和目标

　　2017年，是阜新转型升级的关键之年。我们要看到，以习近平同志为核心的党中央作出对经济形势重大判断、对经济工作重大决策、对经济工作思想方法重大调整，新理念、新思想、新战略引领经济发展新常态。我们要看到，党中央、国务院对新一轮东北振兴精准发力，相继下发了中发7号和国发28号、62号等一系列重要文件，含金量高、操作性强，为我们提供了难得的新机遇。我们要看到，全省经过政治生态修复、“四个着力”落实、“四个驱动”实施，全省经济将进入速度换档、结构调整、动力转换历史节点，我们将迎来转型升级的新局面。我们要看到，阜新业已形成良好的发展基础，一批重点产业布局清晰、呈现了增长态势，一批影响长远的重大项目正在加快孕育、积蓄了巨大潜力，一批新的经济增长点正在加快形成、保持了增长后劲，我们将释放转型发展的新动能。我们要看到，全市广大党员干部群众对转型升级热切期盼、大力支持，团结拼搏、攻坚克难的信心坚定，奋发进取、干事创业的精神饱满，展现出新常态下不服输、不气馁、主动作为的新风貌。只要我们树立强烈的发展意识、机遇意识、责任意识和担当意识，保持战略定力，坚定发展信心，就一定能够凝聚转型升级的强大力量，一定能够创造更加美好的生活。

　　今年政府工作的总体要求是：以党的十八大和十八届三中、四中、五中、六中全会及中央经济工作会议精神为指导，深入贯彻落实习近平总书记系列重要讲话精神，全面贯彻省市党代会和经济工作会部署，统筹推进“五位一体”总体布局和协调推进“四个全面”战略布局，坚持稳中求进工作总基调，牢固树立新发展理念，全面落实“四个着力”要求，以提高经济发展质量和效益为中心，以供给侧结构性改革为主线，以营造良好发展环境为抓手，全面推进经济结构优化升级、发展动力转换升级、社会建设提质升级，努力开创全市经济社会发展新局面。

　　主要预期目标是：地区生产总值增长6.5%；固定资产投资增长15%；一般公共预算收入增长6%；城镇、农村常住居民人均可支配收入分别增长6%。城镇登记失业率控制在4.5%以内，居民消费价格涨幅控制在3% 以内。

　　做好今年的政府工作，我们必须坚持以下四点：一是必须牢固树立新发展理念。坚持创新发展，把创新贯穿政府工作始终；坚持协调发展，提高经济社会发展整体性；坚持绿色发展，打造生态宜居环境；坚持开放发展，营造亲商稳商的良好氛围；坚持共享发展，增加全市人民幸福感和获得感。二是必须全面落实“四个着力”要求。着力完善体制机制，突出深化改革治本之策；着力推进结构调整，抓住结构优化关键之举；着力鼓励创新创业，把握创新驱动决胜之要；着力保障和改善民生，筑牢民生改善稳定之基。三是必须全面推进三个升级。推进经济结构优化升级，加快构筑多元支撑、可持续发展的现代产业体系；推进发展动力转换升级，加快培育转型发展的新优势；推进社会建设提质升级，实现社会和谐稳定发展。四是必须牢牢把握两个红线不能逾越。坚持安全、环保红线原则，坚决落实安全责任，坚决排查治理安全隐患，坚决遏制重特大事故发生；坚决执行环保要求，坚决整改超标排放，坚决查处环保违规行为。

　　三、2017年工作任务

　　（一）着力抓好项目建设，努力扩大有效投资

　　牢牢抓住有效投资的关键作用，把投资作为稳增长、调结构、促转型的重大举措和重要途径。

　　全力以赴推进项目建设。坚持以项目为核心，深入开展“项目年”活动。举全市之力推动大唐煤制天然气项目复工及深加工项目建设，抓好徐工整车、德尔二期、宝厦集成建筑、富国皮革总部搬迁等重点项目全面投产，推进保利生产线智能化改造、双层油罐生产基地、省环保集团环保产业园、邮政集团物流中心等项目尽快开工，实现万达广场、红星国际广场等服务业项目运营，促进辉山肉牛屠宰、天津金地果蔬食品等农产品加工项目全面达效。加快页岩气勘探开发、对外合作步伐。全年新开工投资五千万元以上项目100 个；竣工投产项目100个；围绕新一轮振兴东北三年滚动计划、国家建设专项基金，新增投资亿元以上储备项目100个。围绕国发62号文件，争取一批项目得到上级支持，启动一批重大基础设施项目前期工作，编制完成转型升级行动计划。

　　持之以恒扩大招商合作。开展“招商季”活动，把每个季度都当做招商引资季、项目推进季、落地开工季，逐季度明确任务、落实责任，实行招商工作季考核、季评比，久久为功。聚焦重点地区招商，紧紧抓住京津冀协同发展、“一带一路”建设、与江苏省对口合作、承接长三角地区产业转移、与沈阳区域合作等机遇，深化与国企民企的融合。实施精准招商，重点抓好牵动力大的项目，推进中化集团、国药集团、中石油辽宁公司、北京顺鑫集团等项目合作。实施全领域招商，坚持园区招商、产业链招商、以商招商，坚持大中小项目一起抓、国资民资外资项目一起抓、挂靠配套并购项目一起抓、三次产业项目一起抓，用项目培育企业、发展产业、增加税源。实行市级领导带头招商，全年“走出去”45次，“请进来”50次，签约引进项目100个以上，内资到位增长20%以上。

　　切实优化提升营商环境。主动对标先进地区，开展“服务月”活动，把每个月都作为高效服务月、工作创新月和标准提升月，着力从制度上优化营商环境。打造严格规范的法治环境。动态管理权责清单，及时公布办事流程和收费项目，制定年度行政执法检查计划，并实行审批和公开公告。打造诚实守信的市场环境。加强诚信政府建设，政府在职权范围内作出符合法规的承诺和条件必须按期兑现，不得随意更改。完善社会诚信、企业诚信体系。打造公平公正的竞争环境。依法平等保护本地企业和外地企业的合法权益，企业和群众对损害营商环境的举报投诉，必须在7个工作日内作出答复。打造高效务实的政务环境。认真落实首问负责、限时办结、一次性告知等工作制度，继续推进行政审批事项“两集中、两到位”，“双随机、一公开”监管实现全覆盖，组织开展营商环境专项整治行动。

　　（二）着力深化改革创新，激发更强发展动力

　　坚持以改革创新为动力，用改革的办法破解难题，用创新的方式驱动发展，积极推进重点领域和关键环节改革取得突破。

　　深化供给侧结构性改革。“去产能”方面，关闭退出煤矿2户，支持企业通过技术改造、兼并重组等方式化解产能。“去库存”方面，加快建立房地产平稳健康发展长效机制，合理制定房地产开发计划，开展多种形式的促销活动，商品房销售面积增长12%。“去杠杆”方面，继续防范金融风险，促进金融服务实体经济和创新发展。“降成本”方面，全面落实减税降费、降低要素成本等优惠政策，促进企业降本增效。“补短板”方面，下大力气补齐战略新兴产业、公共服务、县域经济、人才吸引等短板。

　　深化行政管理体制改革。继续推进简政放权、放管结合、优化服务。落实商事制度改革任务，提升工商注册便利化水平。优化行政审批服务，着力推进县区集中行政审批改革。

　　深化企事业单位改革。切实提高交通、城建、旅游3个企业集团的经营水平。继续推进供热资源整合、世通物流园重组等工作。做好中央、省属企业和破产矿“三供一业”承接。全面完成33家经营性事业单位转企改制。稳步推进事业单位分类改革。

　　深化财税、投融资体制改革。继续完善政府预决算约束和管理体系。深化国税、地税征管体制改革。加强政府性债务管理，建立“借、用、还”相统一的政府性债务管理机制。全力以赴防范养老金风险，抓好“收、支、堵”各环节工作，加强基金的管理和运营。充分发挥产业投资引导基金的带动作用。积极推广PPP模式。

　　深化创新驱动战略。大力推进科技创新，与中科院、浙江大学、辽宁工大等院校开展深度合作，重点推进产业园区创新平台建设，充分发挥工程研发中心、院士工作站作用，加快实施高新区、国家大学科技园两大“示范工程”。加快“两化融合”体系建设，推动企业、园区管理生产向数字化、网络化、智能化转变。推进创新型企业培育工程和知识产权强市战略，大力发展高新技术产业。着力集聚人才资源，积极引进各类人才和创新团队。全年开展重大产学研合作20项、技术攻关及产业化10项，培育创新型企业20户。

　　同时，积极推进工业用地市场化配置、基本药物制度、城市公立医院综合改革和国有林场改革等工作。完成农村土地承包经营权确权登记，抓好土地经营权抵押贷款试点和农村承包土地“三权分置”工作。

　　（三）着力壮大工业规模，推动产业向中高端迈进

　　坚持以工业经济为支撑，围绕制造业强市行动纲要和“三四四”工业产业布局，加快工业结构调整优化和产品更新换代，打造阜新特色的产业格局、产品体系。

　　促进重点产业转型升级。进一步明确重点产业发展定位，集中精力推进优势特色产业加快发展，重点在建设牵动性重大项目、培育支撑性骨干企业、开发引领性核心产品等方面下功夫。加快液压装备整机化、智能化发展，提升汽车转向泵、航天液压、装配建筑、整车制造等核心产品和技术水平。加快氟化工“智慧园区”建设，强化与上海有机所、中化集团深度合作，大力培育和发展核心技术、拳头产品，全年产值达到15亿元。推进煤化工产业链向下游延伸，加大副产品加工项目引进力度，煤化工园区完成固定资产投资20亿元。推进农产品加工精深化发展，提高税收贡献份额。推动能源清洁化、板材家居产业一体化、皮革产业高端化取得进展。加快沈彰工业园等产业园区建设步伐。

　　促进新兴产业加速成长。完善电子信息产业的规划定位，加大元器件生产、集成电路、软件开发力度。推进新型材料、生物医药、循环经济、硅砂加工等产业加快发展。

　　促进重点企业做大做强。全力以赴抓好企业运行，继续加大停产半停产企业帮扶力度，充分盘活利用闲置工业厂房和资产，积极为企业解决市场、资金、要素等方面难题。鼓励引导40户重点企业转型升级，推进徐工、鲁花等骨干企业发展壮大，加快远东铜业、华禹冶金等企业达产达效。深化市矿合作，加强与阜矿集团协作融合发展。支持引导产业基础好、成长性好的现有企业通过技术改造、合资合作等方式扩大规模、提质升级。全年实施重大工业技改项目30项。开展规上企业培育计划，规上企业数量稳定在280户以上，规上工业增加值增长7%。大力发展民营经济，抓好民营工业企业“大做强”、“小升规”、“个转企”三项工作。

　　（四）着力实施三年滚动计划，加快发展现代农业

　　坚持以农业供给侧结构性改革为牵动，用工业理念谋划农业发展，提高农业规模化、集约化、标准化和有机化水平。

　　推进现代农业发展。实施新一轮沈阜现代农业三年滚动计划，巩固提升200万亩、再建100万亩，重点在基础设施建设、产业结构调整、新型经营主体培育、农业开放等方面下功夫，提升产出效益和增收水平。当年巩固提升60万亩，新建30万亩。

　　推进农业结构调整。稳定粮食产量40 亿斤，调减玉米面积13.4万亩，扩大经济作物面积。加强粮经饲统筹、种养加一体、农牧果结合发展。大力发展高效农业、精品农业、绿色有机农业、特色农业。设施农业蔬菜产量70万吨。深化畜牧业强市战略，新建改扩建标准化规范化养殖小区20个。

　　推进农业产业化发展。统筹三次产业融合发展，做优粮食、果蔬、油料、肉类、乳品等产业链条，壮大杂粮、花生、生猪、肉驴等产业基地。坚持以资源促招商、以品牌打市场，积极打造地方特色产品品牌和地理标志品牌。继续培育做大一批龙头企业，规模以上农产品加工业产值实现130亿元。

　　推进保障能力提升。新增节水滴灌6万亩。做好永久基本农田划定工作。佛寺水库除险加固工程主体完工。加大新品种、新技术推广力度，增强农机装备能力。巩固农村信息进村入户工作成果，12316热线实现乡镇全覆盖。抓好人工影响天气工作。继续做好第三次全国农业普查。

　　（五）着力提高服务业档次，积极培育新增长点

　　坚持以服务业集聚区建设为重点，促进生产性、生活性服务业发展，培育消费热点，提升服务业发展水平。

　　加快服务业升级。深入推进服务业发展四年行动计划，提高6个省级服务业集聚区建设水平，营业收入增长10%以上。抓好温泉养生、玛瑙文化、民族文化、矿山文化、沙地公园等特色旅游景区开发和产品营销，加快海棠山、黄家沟等旅游景区建设步伐，改造提升旅游大通道。抢抓国家优先支持东北地区企业上市机遇，加快阜新银行等企业上市步伐。积极构建以政府为主体、引导社会广泛参与的中小企业融资担保服务平台，解决中小企业融资难题。加快构建涉农金融机构、农民资金合作互助社等新型农村金融服务平台。

　　加快完善服务业体系。合理控制商贸流通大型市场、专业市场布局，促进现有企业发展壮大、转型升级。发挥区位优势，大力发展冷链物流、快递物流、共同配送等新模式，抓好农产品现代物流体系建设。发展家政服务、社区服务、居家养老服务、健康服务、医疗服务，健全惠及城乡居民的多种形式服务体系。

　　加快培育新业态。实施“互联网+”行动计划，依托我市特色产业基础，着力发展电子商务，完善煤炭、农产品、液压、皮革等领域现货电子交易平台。发展文化创意、服务外包等新兴服务业。

　　加快培育消费热点。促进商贸流通业健康发展，引导企业利用大数据技术推进市场拓展、精准营销和优化服务，满足多层次、多样化消费需求。社会消费品零售总额增长8%。

　　加快培育外贸增长点。实施外贸企业扩户工程，做大骨干企业规模，培育跨境电商平台。全年出口总额2.3亿美元，增长8%。

　　（六）着力加强环境保护治理，建设生态宜居城市

　　坚持以改善环境质量为核心，大力开展环境保护、治理和修复，打造优良人居环境。

　　抓好蓝天碧水青山沃土工程。落实大气、水、土壤污染防治任务，强化总量控制，推进节能减排。拆除燃煤小锅炉233台，加大黄标车及老旧车淘汰力度，推广秸秆综合利用，加快“气化阜新”步伐，确保城市环境空气质量达标率达到80%。加强流域水质监测和污染源监控，严格环境执法监管，保持水环境质量达标。加快细河全流域环境治理步伐，推进流域湿地等生态工程建设。开工建设城市防汛“3+1”工程。建立健全“河长制”。统筹治理、保护和开发，进一步完善海州露天矿及周边环境综合治理规划，提高危害治理水平。完成人工造林15.8万亩、森林抚育3.7万亩。建设好海棠山国家自然保护区和章古台沙地国家森林公园等项目。以农用地和重点行业企业用地为重点，加强土壤污染防治和治理修复工作。

　　抓好基础设施建设。继续配合做好京沈客专阜新段及彰武联接线建设工作。开工建设京沈客专阜新站、阜奈高速公路和辽西北供水阜新配套工程。实施细河生态廊道工程，提升细河城市段生态景观及休闲健身功能。改造供热、供水、供气地下管网50公里以上，做好市政设施维修改造和城区绿化工作。生活垃圾焚烧发电项目投入运行。维修改造农村公路230公里。新建、改造农村电网647公里。

　　抓好城乡建设管理。加快玉龙新城项目开发建设、配套设施完善、人口等要素集聚步伐，全力做好阜新高铁站前板块开发。有效运行数字化城市管理系统，开展市容环境综合整治。积极创建国家级卫生城市，提升城市环境卫生质量。发展城市公共交通，整治交通拥堵。加强住宅小区物业管理。完善重点镇服务功能，建设特色小镇。以农村环境综合整治为抓手，推进宜居乡村建设，争创省级生态乡镇2个、村12个。

　　（七）着力保障改善民生，增强人民群众获得感

　　坚持把人民群众对美好生活的期盼作为我们的目标行动，努力让人民生活更幸福。

　　实施就业创业工程。把就业作为最大的民生，坚决守住就业底线，加强就业和再就业培训，加大对高校毕业生的就业帮扶力度。加快构建创业创新支撑平台，落实优惠政策，激发民间创业热情。全年新增城镇就业1.6 万人。加强和谐劳动关系建设。

　　实施社会保障工程。加快构建城乡一体的社会保障体系，提高城乡居民最低生活保障标准，加快城乡居民基本医保整合。推进社会化养老服务体系建设，鼓励社会力量兴办养老机构。加强大病救助和困难家庭救助，健全特困人员救助供养制度，大力发展慈善和残疾人事业。

　　实施脱贫攻坚工程。全力推进国家扶贫改革试验区建设，加快体制机制创新步伐。深入实施“五个一批”举措，把功夫用到帮助贫困群众解决实际问题上。全年实现建档立卡的3.5万贫困人口脱贫、65个贫困村脱贫“销号”。

　　实施教育满意工程。继续完善城乡学前教育，创建标准化幼儿园20所。推动义务教育均衡发展，完善中考招生制度改革。深化职业教育校企融合发展，培育技术技能型人才。促进民族教育、特殊教育和民办教育健康发展。改造薄弱学校11所，维修改造城乡校舍10万平方米。

　　实施医疗健康工程。深入推进健康阜新建设，强化基层卫生服务、公共卫生服务体系建设，做好重点传染病、慢性病防控工作。提高全市临床重点专科建设水平。加快蒙医蒙药事业发展。

　　实施生命接力工程。倡导遗体、器官、造血干细胞捐献和无偿献血。

　　实施文化精品工程。加强“世界玛瑙之都”城市文化品牌宣传和推介。积极备战省第十届艺术节。繁荣文化艺术创作，丰富群众文化艺术生活。

　　实施体育惠民工程。巩固“全国全民健身示范市”成果，提高竞技体育水平。举办第三届篮球节。

　　实施人居改善工程。推进独立工矿区改造、采沉区治理及城市危楼改造。开工棚户区改造1291套，改造农村危房1355户。

　　实施平安阜新工程。全面落实安全生产责任制，强化安全生产监管，推动安全生产形势持续稳定。提高食品药品安全管理水平。进一步完善社会治安防控体系，确保社会安全和谐稳定。

　　强化信访工作责任落实。不断提高双拥工作水平。重视和支持民族地区发展。依法加强对宗教事务的管理。

　　（八）着力提升推动发展的能力水平，切实加强政府自身建设

　　全面履行从严治党主体责任，坚决做到忠诚、干净、担当，努力建设法治政府、诚信政府、服务政府、廉洁政府。

　　坚持忠诚履职。牢固树立“四个意识”，坚决维护以习近平同志为核心的党中央权威和党中央集中统一领导，真正做到思想上深刻认同核心、政治上坚决维护核心、组织上自觉服从核心、感情上坚决拥戴核心、行动上坚定紧跟核心。牢固树立抓发展必须抓党建的理念，把抓好党建作为最大的政绩，坚决履行好“一岗双责”，以党建统领各项工作。坚决维护省委、市委的权威，坚决贯彻执行党中央、国务院、省委、省政府和市委的各项决策部署，坚决维护良好的政治生态。

　　坚持干净干事。推进依法行政，切实加强制度建设，规范行政权力运行和监督，牢牢扎紧制度的笼子。自觉接受人大法律监督、政协民主监督，进一步提高建议提案办结率、落实率，注重听取各民主党派、工商联、无党派人士和人民团体的意见建议。推进政府职能转变，把该管的管到位、该放的放到位。加强审计监督、行政监察和执纪问责，强化政务公开，坚决纠正损害群众利益的不正之风。坚持廉洁从政，严格执行中央八项规定和省市委十项规定及细则，持之以恒加强作风建设，牢固树立“过紧日子”思想，厉行节约、反对浪费，深入推进反腐倡廉取得新成效。

　　坚持担当尽责。抓住“想干事、敢干事”这两个关键点，着力破除政府系统部分干部不作为、不担当、不适应的问题，进一步提高履职能力和服务水平。增强责任意识，崇尚实干，狠抓落实，实行工作细化、量化、实化管理，雷厉风行抓落实，精益求精抓落实，坚持不懈抓落实。强化担当精神，面对矛盾敢于迎难而上，面对问题善于精准解决，面对困境勇于突破创新。建立健全奖励表彰、工作业绩与要素支持挂钩、督察检查、绩效考核、容错纠错等机制，让广大干部群众干事创业的激情竞相迸发，让阜新大地蓬勃发展的动力充分释放。

　　各位代表，阜新转型升级的光荣使命，鼓舞我们开拓进取；新一轮振兴发展的重大机遇，鞭策我们奋发有为；全市人民的梦想福祉，激励我们勇往直前。

　　各位代表，时代赋予重任，拼搏铸就辉煌。让我们在市委的领导下，在市人大和市政协的监督支持下，紧紧团结和依靠全市各族人民，勇于担当，真抓实干，努力开创阜新转型升级新局面，以优异成绩迎接党的十九大胜利召开！
